# Memoir on the Loss of the Teeth

**Published:** 1856-04

**Authors:** 

**Affiliations:** Paris


### ARTICLE XXII.

*Memoir on the Loss of the Teeth.* By Dr. Rossi, Paris, 1852.

A great many books have been written on the teeth. But few have written earnestly and conscientiously. The greater number in our epoch have had no other aim than to display false erudition, often borrowed or paid for, or what is still more deplorable, to cast their name noisily before the public. As for the last mentioned, their desire to usurp a place among men of merit, is not even an excuse.

It is not our intention to write a bibliographical and critical work; we will quote nobody nor discuss any opinion. We wish to sketch a complete and rational theory, in which the facts are all of equal value, and explain themselves. We do not pretend to have discovered all, or to have invented all. Let those who think they have something to claim, reclaim it.

Our theory is an absolute affirmation, because it is based upon truth.

We will be brief, because we have not twenty varieties of a fantastic disease called *caries*, to describe. It has also appeared to us unnecessary to examine the value of the pathological doctrine of which our venerable member, Doctor Duval, has been a promoter. It begins to lose many of its partisans, and the strong faith in which it was proclaimed in our youth, has already become much enfeebled. Observers of the present day are in a state of doubt, and we now hear eighteen or twenty kinds of caries spoken of only by some new and courageous compiler, who enumerates, as truths, the speculations of past ages. We esteem the author of *Dentiste de la jeunesse*, without always sharing his opinions.

He may feel consoled, however, even if his doctrines begin to be severely shaken. During a period of forty years, numberless writers have been supplied by him. Books, pamphlets, articles for dictionaries, treatises on surgery, &c., have copied and re-copied him to satiety. He might often have laid claim to right of authorship, if a juster law had existed.

We do not count upon the same success. Truth is our only support, and we know how importunate it is sometimes. And, finally, certain men, still puffed up by their collegiate pride, consider themselves as *the doctors* of this protean disease, styled *caries.* To descend from this scientific throne requires a denial which is not easy for human weakness to bear. However this may be, we will not be led by this spirit. The spread of truth, even if slow and obstructed in its course, is not less sure, and we have among us honest hearts and noble spirits, who always accept a ray of light, from however humble and lowly the source.

Lastly, a consideration of the utmost importance prompts us. False doctrines often lead to fatal consequences. The means proposed to be employed to overcome the loss of the teeth, flow from the opinions, false or true, which are held upon this subject. It is clearly evident then, how important it is that the truth be known and accepted, in order that error lead no more to injurious operations and to inefficacious treatments.

We shall feel sufficiently rewarded if we have contributed in any measure, however small it may be, toward this desirable result.

*Proposition.—Assertion.—The change in the teeth, improperly called* CARIES, *is a chemical phenomenon, the causes of which are all, and always, apart from life.*

Philosophically expressed, a change in a tissue has an essential and inevitable cause, a modification of nutrition.

In order that this modification of nutrition be possible, the tissue must possess nerves and vessels.

But, the osseous tissue of the teeth receives neither vessels nor nerves.

Therefore the change in the osseous tissue of the teeth, so singularly called CARIES, is not a modification of nutrition, a disease.

In an animal, only organic or inorganic phenomena can take place.

But the change of tissue which occupies our attention, cannot be an organic phenomenon, since it does not result from a modification of nutrition.

Therefore, the alteration in the tissue of the teeth, improperly called *caries*, is an organic phenomenon, or in other words: *This change is a chemical phenomenon, in which the causes are all and always apart from life*, which is the assertion of this proposition.

*Exposition.*—§ A.—The osseous tissue of teeth is never diseased.

The destruction of the tooth is due to *a single cause*—the action of one or several acids.

*These acids exist,*

1st. In the fluids secreted by the subject.

2d. They are produced by elements coming from without, in food, or already formed, directly introduced into the mouth.

Acids secreted by the subject, are found in the buccal mucus, in the saliva and gastric juice. Their quality and quantity depend upon the constitution of the subject, upon a natural though transient state, (pregnancy, lactation,) upon certain diseases to which one may be, for a time, subjected.

The acids produced from elements brought from without, arise from the decomposition of food in every place where it can lodge. This fermentation is putrid or acid. Certain food furnish more active and concentrated acids than other. Acids introduced into the mouth that are already formed, are generally in drinks or medicines.

§ B.—The decomposition of teeth by acids is considerably favored by predisposing causes, which we will enumerate:

1st. By the quality of the tissue of the teeth.

2d. By the form of the teeth.

3d. By the arrangement of the teeth.

4th. By traumatic lesions.

### DESTRUCTION OF THE TEETH.

### ONE CAUSE.

### ONE OR MORE ACIDS.

- Secreted by the subject in
  - The buccal mucus.
  - The saliva.
  - The gastric juice.
- Formed by elements, independent of the subject
  - and resulting from the decomposition of food.
    - putrid substances (meats, etc.)
    - acetic substances (milk, etc.)
  - or existing before their introduction,
    - in certain drinks (cider, etc.)
    - in certain medicines (sulphuric lemonade, etc.)
- Favored by particular condition. (predisposing causes.)
  - 1st. Quality of the tissue of the teeth.
  - 2d. Form of the teeth.
  - 3d. Arrangement of the teeth.
  - 4th. Traumatic lesions.

[*Dent. Rec.*

